# Basic Biology of Hypoxic Responses Mediated by the Transcription Factor HIFs and Its Implication for Medicine

**DOI:** 10.3390/biomedicines8020032

**Published:** 2020-02-13

**Authors:** Kiichi Hirota

**Affiliations:** Department of Human Stress Response Science, Institute of Biomedical Science, Kansai Medical University, Hirakata, Osaka 573-1010, Japan; khirota-kyt@umin.ac.jp; Tel.: +81-72-804-2526

**Keywords:** hypoxia, transcription factor, hypoxia-inducible factor 1, HIF-1, hypoxia sensing

## Abstract

Oxygen (O_2_) is essential for human life. Molecular oxygen is vital for the production of adenosine triphosphate (ATP) in human cells. O_2_ deficiency leads to a reduction in the energy levels that are required to maintain biological functions. O_2_ acts as the final acceptor of electrons during oxidative phosphorylation, a series of ATP synthesis reactions that occur in conjunction with the electron transport system in mitochondria. Persistent O_2_ deficiency may cause death due to malfunctioning biological processes. The above account summarizes the classic view of oxygen. However, this classic view has been reviewed over the last two decades. Although O_2_ is essential for life, higher organisms such as mammals are unable to biosynthesize molecular O_2_ in the body. Because the multiple organs of higher organisms are constantly exposed to the risk of “O_2_ deficiency,” living organisms have evolved elaborate strategies to respond to hypoxia. In this review, I will describe the system that governs oxygen homeostasis in the living body from the point-of-view of the transcription factor hypoxia-inducible factor (HIF).

## 1. Introduction

The theory of phlogiston was proposed before the discovery of O_2_ [1,2]. It was believed that substances burned in air were rich in phlogiston. The fact that combustion soon ceased in an enclosed space was taken as clear-cut evidence for air having the capacity to absorb only a finite amount of phlogiston [3,4]. When air became completely “phlogisticated,” it was no longer able to support the combustion of any material. This led to the concept that “burning” was a process, which released a substance termed phlogiston. According to this theory, fresh air does not contain phlogiston. Thus, oxygen was “discovered” as a so-called anti-phlogiston substance. Thus, from the very beginning of its discovery, oxygen was considered because of its deficiency [3]. Unlike gas molecules such as nitric oxide (NO), carbon monoxide (CO) and hydrogen sulfide (H_2_S), oxygen is not produced in vivo and easily becomes “deficient” when supply is discontinued or diminished. In clinical settings, hypoxemia, defined as a drop in the partial pressure of O_2_ in blood, leads to hypoxia. However, according to experimental as well as clinical evidence, the concept of a “normal” blood O_2_ concentration cannot be unitarily defined. Hypoxia is conceptually defined as a lack of O_2_ in the whole body or specific tissues/organs. Under hypoxic conditions, O_2_ metabolism is suppressed. Usually, a decrease in absolute O_2_ supply is indicated, but in clinical settings, a negative mismatch between O_2_ supply to tissues/cells and O_2_ demand is considered as a hypoxic state. When O_2_ metabolism is suppressed, a biological response resembling an hypoxic response may be induced even when O_2_ is amply available. When O_2_ consumption rises beyond the limits of cardiopulmonary capacity for supplying O_2_, or when an organ is exposed to inflammatory mediators due to sepsis or conditions involving disturbed O_2_ utilization due to drugs or mitochondrial disorders, a response resembling a hypoxic state is observed at the tissue/cell level. On the other hand, erythropoietin (EPO)-producing cells (REP cells), believed to be present in kidney stroma, may sense discrepancies between supply of, and demand for, oxygen and regulate erythrocyte production by modulating EPO secretion. EPO concentrations change constantly. Evidently, physiological “hypoxic regions” exist and play an important role in signal transduction in vivo. Reactive oxygen species (ROS) generated by oxygen exert toxic effects, but also play an essential role as secondary messengers in intracellular signal transduction. Thus, O_2_ plays a role in both energy production and signal transduction in the body. This field of study may be termed investigative hypoxia biology. The partial pressure of O_2_ in the cells is kept within a relatively narrow range. In humans, the O_2_ partial pressure in the alveoli is about 110 mmHg, and the partial pressure of O_2_ in the heart, kidney and brain is partially about 20 mmHg. The O_2_ partial pressure of all cells is determined by the supply and consumption of O_2_. Deviations such as hypoxia and hyperoxia trigger an adaptive response to maintain O_2_ homeostasis at the cellular level. For the proper function and regulation of these systems, a harmonious expression of thousands of genes is probably required. In this review, I will describe the system which govern the O_2_ homeostasis in the living body from the point of view of the transcription factors hypoxia-inducible factor (HIF).

## 2. Hypoxic Conditions in the Living Body

**Figure 1:**
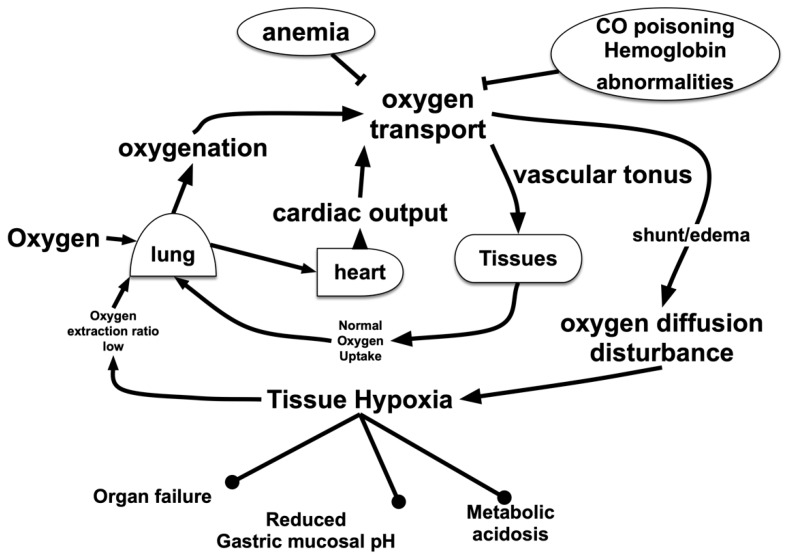
Oxygen (O_2_) flow in the living body. O_2_ is carried from the lungs to peripheral organs and tissues by red blood cells through blood vessels. Impaired oxygenation due to lung injury, disorders in transport due to anemia or blood clots and/or disturbance of transition from blood vessels to cells due to interstitial edema result in tissue hypoxia. In addition, O_2_-related metabolic disturbances due to suppression of O_2_ utilization, such as drug-induced mitochondrial suppression, might have an effect on the living body, which is equivalent to that of hypoxia.

Hypoxic conditions in the living body in which O_2_ metabolism is suppressed may be classified according to the underlying mechanism as follows [5,6,7] (Figure 1).

### 2.1. Hypoxic Hypoxia

Hypoxemia is a state of reduced oxygen partial pressure in the blood, the onset mechanisms of which are a low O_2_ partial pressure (caused by high altitudes or enclosed spaces), alveolar hypoventilation (drug effects and sleep apnea), loss of gas exchange efficiency in alveoli (pulmonary edema and acute lung injury), presence of pulmonary or intra-organic shunt (liver cirrhosis, congenital malformation) and ventilation decrease of the blood flow ratio (body position, artificial respiration). Hypoxemia can be assessed via a pulse oximeter even without drawing blood. Arterial blood gas analyses have been performed under extreme conditions (e.g., at atmospheric pressure of 253 mmHg, found at altitudes of 8400 m above the Everest summit, the PiO_2_ was 43.1 mmHg; the average value of four individuals) [8,9,10].

### 2.2. Hypemic Hypoxia

Apart from being due to hypoxemia, the O_2_ transport capacity of blood may decline for various other reasons, such as carbon monoxide poisoning [11,12], methemoglobinemia [13,14], congenital hemoglobin disorders [15,16,17] and impairment of O_2_ transport capacity to tissues/organs. O_2_ partial pressure in the blood is maintained. It may be determined by analyzing blood via a CO-oximeter. Anemia, in which the oxygen-carrying capacity is reduced due to a decrease in the red blood cell count and hemoglobin levels, is also included in this category. Hematopoietic disorders or iron deficiency may act as an onset mechanism for anemia. Anemia can be diagnosed via blood tests. Few studies have explicitly elucidated the actual impact of anemia, even though hemoglobin in red blood cells is known to be responsible for the oxygen-transporting ability of blood. We examined the difference in EPO expression between hypoxemia in 12.9-week-old C57BL mice exposed to a 10% oxygen environment (approximately 82% SpO_2_) and an anemia group (200 μL of blood removed from the orbital vein for a decrease in the hematocrit from 44% to 33%) [18,19]. The plasma erythropoietin concentration was measured via ELISA 3 h later. The hypoxemia group contained 1000 pg/mL and the anemic group contained 1400 pg/mL, compared with 50 pg/mL for the control group. Thus, the anemic state induces a robust hypoxia-inducible gene response in not only the kidneys but also in the brain and the liver.

### 2.3. Tissue Hypoperfusion and Ischemia

Ischemia occurs because of low blood pressure, a thrombus or an embolus. Ischemia (hypoxia + low glucose + low amino acid) and hypoxia elicit completely different responses at the cellular level. Enhanced activation of hypoxia-inducible factor 1 was observed under hypoxic conditions (a 1% oxygen environment). However, activation was drastically attenuated under conditions that imitated ischemia (low oxygen + low glucose + low amino acid). Failure of blood flow in the limbs and skin can be easily detected, but visceral blood flow failure can only be assessed via ultrasonography, contrast CT or a similar technique [20,21].

### 2.4. Tissue Oxygen Metabolism Disorder

Even if O_2_ supply is maintained above a certain level, O_2_ burden may occur when O_2_ consumption in the tissues/organs increases. Such a disease state may be caused by tissue O_2_ metabolism disorder. Conditions that suppress O_2_ metabolism in cells, such as cytotoxicity, mitochondrial suppression via therapeutic drugs or cyanide poisoning [22,23], biguanide-induced lactate acidosis [24,25] and propofol infusion syndrome [26,27], are classified in this category. However, oxygen metabolism disorder cannot be accurately diagnosed in real-time prior to organ dysfunction or some such effect becoming apparent. Only estimation methods based on the concentration of marker substances, such as lactic acid, are effective.

## 3. Hypoxia-Sensing Mechanisms

**Figure 2:**
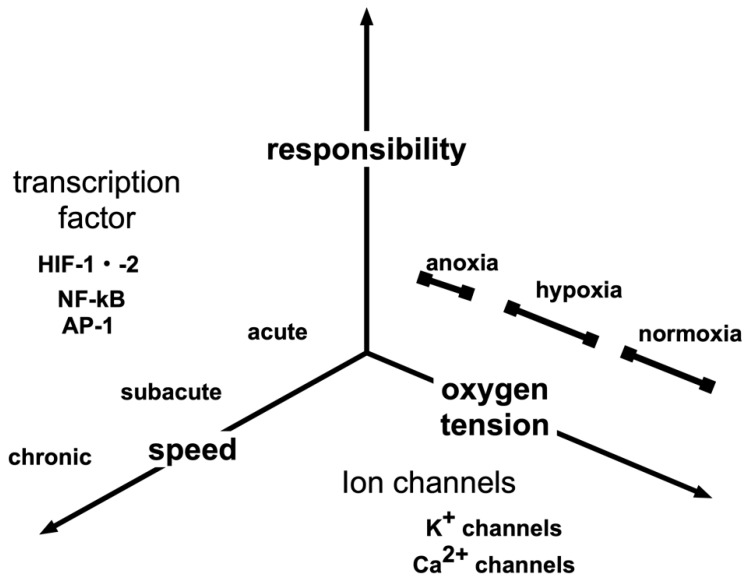
Various hypoxic responses. The hypoxic response of a living body may be classified in various ways according to the presence or absence of the gene response, rapidity and oxygen partial pressure that induces the response. In addition to HIF-1, transcription factors responsible for hypoxic responses include NF-κB and AP-1, which are master transcription factors for inflammatory responses. Hypoxic responses may vary from those that are triggered quickly, within minutes, such as hypoxic pulmonary vasospasm responses, to those involving several hours, such as erythropoietin induction. In addition, high altitude adaptation may occur over a few weeks.

Molecular O_2_ is essential for the development and growth of multicellular organisms. Mammals have evolved a sophisticated physiological network, which involves the capture, binding, transport, and delivery of molecular oxygen in order to maintain O_2_ homeostasis at the tissue level. A critical aspect of this network is the ability to sense and respond to low O_2_ conditions (Figure 2).

**Figure 3:**
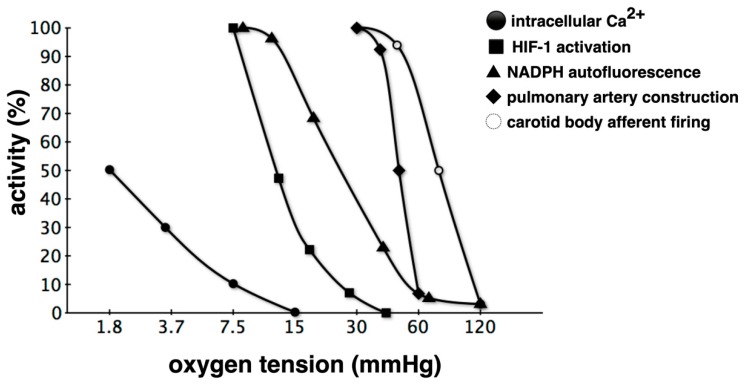
Relationship between oxygen tension and activities of hypoxic responses. Classification based on the partial pressure of O_2_ that triggers a response is also possible. Hypoxic pulmonary vasospasm reactions begin at a higher oxygen partial pressure compared to HIF-1 activation, and intracellular Ca^2+^ elevation is mostly observed under anoxic conditions. Such varying hypoxic responses are probably due to differences in the corresponding O_2_ partial pressure sensing mechanisms [28,31,34,41,42,43,44]. Hypoxia detected via several sensors is triggered as a hypoxic response through various effectors, but crosstalk occurs during the transduction of such signals. For example, some voltage-dependent K^+^ channel subtypes are regulated by HIF-1. NAPDH: nicotinamide adenine dinucleotide phosphate.

Hypoxic responses by the body are not uniform. Therefore, hypoxic sensing mechanisms are not unique (Figure 3). When a biological system senses a reduction in arterial oxygen partial pressure at the carotid and aorta bodies, signals are transmitted to the respiratory center in response [28,29]. Glomus type I cells in the carotid and aortic bodies act as sensing cells of this system. Type I glomus cells depolarize when O_2_ partial pressure decreases. This opens voltage-gated calcium channels resulting in a flow of calcium ions into the cytoplasm, which causes exocytosis of vesicles containing neurotransmitters [30,31,32]. In the event of hypoxic pulmonary artery contraction, sensor cells detect O_2_ tension [33,34,35]. Renal EPO-producing (REP) cells in kidney stroma sense O_2_ concentrations in renal arteries [36,37]. Thus, each of these hypoxic responses is associated with a sensing mechanism that may be defined via molecular biology. However, there is no consensus on whether molecular mechanisms underlying ion channel-based hypoxia signaling represent a universal strategy. It is not known whether similar or different O_2_-sensing mechanisms are utilized during acute and chronic responses in depolarizable and nondepolarizable cells. A priori, one may postulate a variety of mechanisms by which cells may sense oxygen concentration levels. In the simplest model, the sensor binds oxygen directly, causing the fraction of sensor molecules bound to ligands to decline with the oxygen concentration. The bacterium, *Rhizobium meliloti*, exhibits a two-component signaling system consisting of FixL, a hemoprotein kinase that is active in the deoxy state, and FixJ, a transcription factor that becomes active when phosphorylated by FixL [38,39]. Hemoglobin also acts as an O_2_ sensor. The 3D structure of hemoglobin allosterically changes in response to O_2_ tension. Although there is a lack of experimental support for a ligand model, it is possible that mammalian O_2_ sensing involves molecular interactions with one or more hemoproteins. Besides heme, iron-sulfur clusters represent another intracellular target for O_2_. In the presence of O_2_, iron regulatory protein 2 is degraded via iron-sulfur cluster formation [40].

Reportedly, mice display a mechanism by which the odor receptor, *Olfr78*, is able to sense an increase in lactic acid due to a decrease in oxygen, resulting in the excitation of the glomus I cells of arteriolar bodies [32,45]. A hypoxic response may be categorized by the threshold at which the reaction is triggered as well as by the mechanism of the reaction or the speed of the reaction. These differences may be due to differences in oxygen partial pressure sensors.

Although research using classical biochemical methods has a long history, molecular biology methods were introduced only in the 1990s. cDNA cloning of hypoxia-inducible factor 1 (HIF-1) became an important turning point in modern oxygen biology [46].

This review will focus on the biology of hypoxia-inducible factors in relationship with medicine.

## 4. Hypoxia-Inducible Factors

Evidently, hypoxia is not only sensed by speciallized tissues and organelles, such as the carotid body and pulmonary artery smooth muscle, but also by all nucleated cells. A cellular factor that plays an essential role in hypoxia-elicited gene responses has been identified. It is the transcription factor hypoxia-inducible factor 1 (HIF-1). HIF-1 was originally isolated as the factor responsible for inducting erythropoietin under hypoxic conditions [47,48,49,50,51]. HIF-1 is a heterodimer consisting of an alpha subunit (HIF-1α) and a beta subunit (HIF-1β, ARNT) [50]. When cells sense hypoxia and HIF-1 is activated, HIF-1 recognizes and binds to a special sequence (hypoxia response element) in the regulatory domain of the target genes, thereby activating or silencing the transcription of an entire line of genes [48]. Early studies using cell lines indicate that HIF-1 increases erythrocyte production via erythropoietin, vasculogenesis via vascular endothelial cell growth factor and glycolysis via a series of glycolytic enzymes. Although HIF-1 was originally isolated as the factor responsible for EPO induction, it has in the meantime been shown that EPO is a target of HIF-2 [52,53]. Consider metabolism as an example as well [54,55,56,57]. When oxygen concentrations are normal, mitochondria maintain oxidative phosphorylation at a level that is sufficient to produce ATP (aerobic metabolism) efficiently. However in environments that are low in oxygen, metabolism shifts to glycolysis (anaerobic metabolism). HIF-1 activates a gene set encoding the enzymes responsible for glycolysis (9 of the 12 glycolytic enzymes regulate HIF-1 expression), and conversely, another gene set that suppresses oxidative phosphorylation to facilitate metabolic reprogramming [58,59]. The body attempts to adapt more to hypoxic stress by mobilizing a large number of genes via HIF-1 at cellular, tissue, organ, and systemic levels to maintain cellular energy, blood flow, and oxygen transportation competence. A comprehensive analysis of gene expression revealed that hypoxia-inducible expression of over 5000 genes (at least 20% of all genes) was controlled by HIF-1 [60].

There are three isoforms of HIF: HIF-1, HIF-2, and HIF-3. The three types of HIF-α subunits have a basic helix–loop–helix (bHLH) region at the N-terminus and a Per-ARNT-Sim homology (PAS). Among the HIF-α subunits, HIF-1α and HIF-2α are N-terminal transactivation domain (N-TAD) and a C-terminal transactivation domain (C-TAD). These two subtypes control the expression of common sets of genes and expression of subtype-specific sets of genes due to the differences in post-translational modifications and the expression among cells and tissues [61,62]. The subtype-specific controls confer the diversity of the hypoxic response. On the other hand, HIF-3α lacks the component of C-TAD.

HIF-1α and HIF-2α have 48% homology at the amino acid sequence level [61]. Homology in the functional domains is higher. The topic is how HIF-1 and HIF-2, which have high homology to the primary structure, play differential roles in each cell and body.

HIF-1 was originally isolated from liver and cervical cancer cell lines as a transcription factor responsible for the induction of EPO expression. According to genetic engineering analysis using mice thereafter, HIF-2 but not HIF-1 is a transcription factor that mainly works in EPO-producing cells in the stroma of the kidney [37,63,64]. The genetic variation of EPAS1 (HIF-2α) has been reported by pedigree analysis of human familial polycythemia [52]. Although HIFs are thought to play an important role in cancer progression, the role of privileged HIF-2 in renal and adrenal cancer has been elucidated [61]. On the other hand, molecules closely related to energy metabolism often depend on HIF-1 for the regulation of expression [61].

The ontology of the differences is ensured by various molecular mechanisms. Although HIF-1α and HIF-2α have considerable homology, there is directivity for post-translational modification of proteins and binding to DNA, and each has a specific set of genes. There is also an explanation that the transcription of HIF-1α and HIF-2α is regulated in organs and tissues, and there is a bias in expression in certain cells [61]. A HIF-2α-specific inhibitor PT2385 has also been developed and is under clinical trial as a therapeutic agent for renal cancer [65,66,67,68].

**Figure 4:**
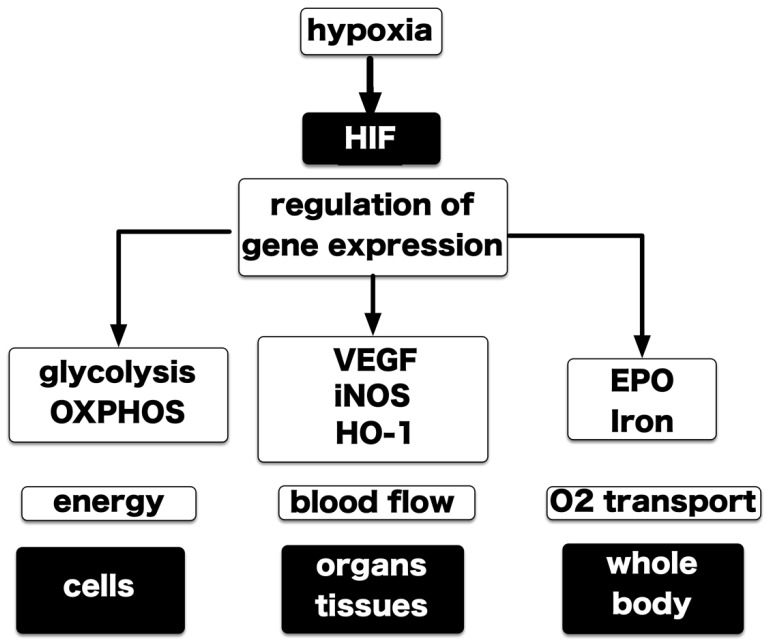
The network of transcription factors and hypoxic responses. Gene responses that depend on HIF-1 and HIF-2 (HIFs) are an important element of the response to low-oxygen by the living body and form an elaborate network. Responses are generated at the cellular, tissue, organ or whole-body level. At each level, a low oxygen response appears as the sum of the responses of individual cells. HIFs have been identified as a key transcription factor in this network. HIFs are present in all nucleated cells, and its activation depends on the partial pressure of oxygen to which cells are exposed. However, activation may be modified by factors specific to cells and tissues. iNOS: inducible nitric oxide synthase; HO‒1: heme oxygenase 1.

O_2_ supply to cells takes place via simple diffusion. However, advanced multicellular organisms that are anatomically complex are specialized in a manner in which all cells may receive sufficient oxygenation. The respiratory system consists of the lung, the diaphragm, muscles aiding respiration, and neuroepithelial cells that sense the partial pressure of O_2_ to maintain O_2_ transfer to hemoglobin in red blood cells. The circulatory system is composed of red blood cells as O_2_ carriers, the heart as the carrier engine and blood vessels as the carrier. For proper development and maintenance of these systems, coordinated expression of several thousand genes is required. HIF-1 is a molecule that changes its activity specifically to suit conditions involving hypoxia and participates in the expression of many hypoxia-inducible genes [54] (Figure 4).

## 5. Central Dogma of the Molecular Mechanism of HIF-1 Activation

Although preliminary studies indicated that HIF-1 contained non-heme iron [69,70], the results of these studies could not be reproduced. HIF-1α is a basic helix–loop–helix PAS protein, wherein its PAS domain is extensively utilized for O_2_ and redox sensing by archaea bacteria, such as FixL [39,71], suggesting that HIF-1 activity may be directly affected by changes in O_2_ concentration. However, these hypotheses have been rejected.

**Figure 5:**
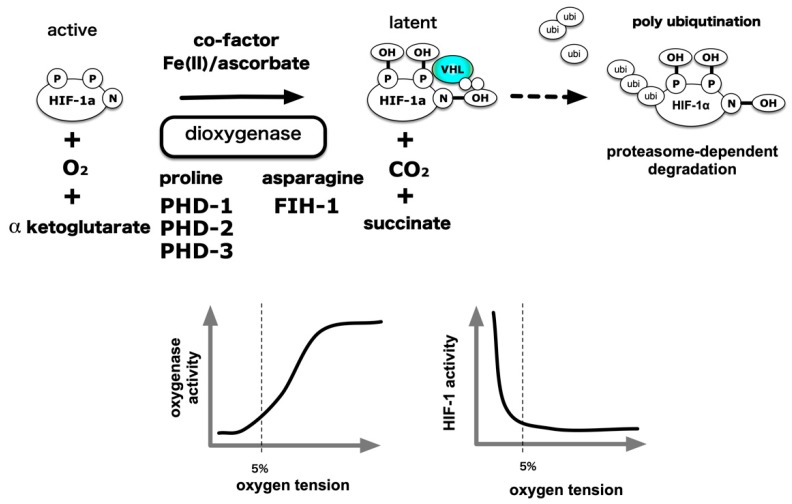
Molecular mechanism of hypoxia-elicited HIFs activation. Mainstream HIF-1 and HIF-2 activation is due to the hydroxylation reaction by the prolyl hydroxylase domain (PHD) protein, which is a HIF-α prolyl hydroxylase and factor inhibiting HIF-1 (FIH-1) protein, which is HIF-α asparaginyl hydroxylase. O_2_ acts as a substrate for these enzymatic reactions. Thus, a decrease in O_2_ concentration results in a decrease in enzymatic reactions, resulting in an accumulation of HIF-α proteins in the cell, which in turn activates it as a transcription factor, thereby resulting in gene expression. Intracellular processes that influence this reaction can be regulators of HIF-1 activity independent of O_2_ tension. In addition, certain intracellular signals can increase the translation of HIF-1α protein and promote HIF-1 activation. P: Proline, N: Asparagine VHL: von Hippel–Lindau.

An outline of the oxygen partial pressure sensing mechanism has been presented in order to explain molecular mechanisms underlying HIF-1 activation under hypoxic conditions [72,73,74,75]. The HIF-1α protein is translated from messenger RNA and undergoes modification via oxygenases (hydroxylases). In the presence of sufficient oxygen, its proline residues are hydroxylated and HIF-1α ubiquitination modification is dependent on hydroxylation of its proline residue by the E3 ligase, VHL, because of which the modified, poly-ubiquitinated HIF-1α protein is transported to the proteasome, an intracellular protein destroying entity, and degraded. On the other hand, independent of protein stabilization, the ability of HIF-1α to activate transcription is also controlled by hydroxylation modification of the asparagine residue [76,77,78]. Hydroxylated HIF-1α is inactive as a transcription factor. Under hypoxic conditions, molecular oxygen is deficient as a substrate for this enzyme reaction, and because of which hydroxylation of proline residues and asparagine residues is suppressed, protein destruction is reduced, and HIF-1α accumulates in the cell. It has been shown that HIF-1α, HIF-2α, and HIF-1β enter into the nucleus by the action of nuclear transport receptors importins α/β. HIF-1α,-1β and HIF-2α are binding to importin α1, α3, α5, and α7 [79,80,81]. It is also reported that importin α4 and α7 is involved in the nuclear transport of the HIF-1a subunit [82]. In the nucleus, HIF-α dimerizes with HIF-1β and exerts an activity as a transcription factor. Two types of dioxygenases play an essential role in this scheme. Proline hydroxylases have three types of isozymes, which are prolyl hydroxylase domains (PHD) 1 to 3 [55,83]. Asparagine residue hydroxylase was isolated as a factor inhibiting HIF (FIH)-1 [84,85]. Subsequent analyses demonstrated that these enzymes catalyze the oxidation of proline or asparagine residues, which require molecular oxygen and α-ketoglutarate as a substrate. Divalent iron (Fe^2+^) and ascorbic acid act as essential co-factors for such enzymatic activity. Thus, a signal indicating a decrease in the partial pressure of the oxygen substrate is converted to a decrease in the activity of the oxygenated enzyme. As a result, the proportion of the hydroxylated product among newly formed HIF-1α decreases in association with VHL and decreases ubiquitination [86]. The “Central dogma” declaring the existence of a balance between protein destruction and neogenesis shifts and HIF-1α protein accumulation in the cell, was established in 2001. A particular finding in this field was awarded the Nobel Prize in Physiology or Medicine in 2019 [46,74] (Figure 5).

Hydroxyl modification activity was examined using recombinant PHD1 prepared in vitro by an in vitro transcription-translation methodology, and the activity of PHD1 was suppressed from 21% to 0% O_2_ conditions following a decrease in oxygen partial pressure [83]. Using the 20-amino acid polypeptide as a substrate for recombinant protein increase in insect cells, it was found that the *Km* of PHDs for oxygen as a substrate was in the range of 230–250 µM, while the Km of FIH-1 was 90 µM [87,88] (Table 1). Physiologically, the partial pressure of oxygen in human alveoli is slightly less than 100 mmHg, or approximately 13%, at 1 atmosphere. It was reported to be about 40–20 mmHg (5%–3%) in the interstitium, which causes diffusion from the capillaries through the arteries, where the intracellular partial pressure further decreases to 20–10 mmHg (3%–1.3%). Another study indicates that the established hepatoma cell line HepG2 cells derived from human hepatocellular carcinoma are exposed to an oxygen partial pressure of 2 mmHg or less, under culture conditions of 20% oxygen [89]. O_2_ solubility in pure water at 20 °C, and 100, 20 and 2 mmHg are equivalent to approximately 36, 7.2 and 0.7 µM, respectively. Considering the above evidence, and the intracellular concentration of α-ketoglutarate, ascorbic acid and divalent iron, the activity of PHD is continuously suppressed from 20% O_2_ to 0% anoxia. The evidence goes against the dogma that HIF-1α hydroxylase is a bona fide hypoxic sensor. When oxygen, the final electron acceptor in the electron transport system, decreases in a low oxygen environment (1%–5% O_2_), the electrons that have lost the acceptor react with oxygen without passing through the cytochrome C-respiratory chain enzyme complex to generate superoxide (O^2−^). O^2−^ is converted to hydrogen peroxide by Mn-SOD or Cu/Zn SOD present in mitochondria and goes out of mitochondria to oxidize divalent irons into trivalent irons, inhibiting HIF-α-hydroxylase enzymatic activity [90,91,92]. Based on the above experimental facts, there are claims that the bona fide hypoxia sensor is a mitochondrion, and that the HIF-α hydroxylation system is an execution system located downstream of mitochondria [93].

HIF-α subunits are post-translationally regulated by the hydroxylation of prolines (catalyzed by PHDs) and the hydroxylation of asparagine (catalyzed by FIH-1). In addition to hydroxylation, both HIF-1α and HIF-2α are subject to a range of distinct, O2-dependent and independent post-translational modifications. Early work showed that both HIF-1α and HIF-2α are phosphorylated [94,95]. HIF-1α is phosphorylated by MAPK [94], casein kinase 1 (CK1) [96], ataxia telangiectasia mutated (ATM) [97], glycogen synthase kinase 3(GSK3) [98], Polo-like kinase 3 (Plk3) [99] and protein kinase A (PKA) [100,101] under hypoxic and normoxic conditions. Phosphorylation affects HIF-α protein expression through regulation of translation and regulation of intracellular stability. In addition, it modulates HIF activity by altering intracellular localization and controlling transcriptional activation. HIF-2α is also a substrate of these kinases [95,102,103].

The activity of HIF-α proteins is also modulated by sirtuins, a family of redox-sensitive, NAD+-dependent deacetylases and/or ADP-ribosyltransferases. Mammalian cells express a family of sirtuins (SIRT1–7) that regulate complex changes in gene expression, metabolism and the cellular redox status [104]. SIRT1 forms a complex with HIF-2α and deacetylates conserved lysine residues in the N-TAD to enhance HIF-2α transcriptional activity [105]. SIRT1 was also reported to deacetylate lysine residues in HIF-1α, which resulted in HIF-1α transcriptional repression [106]. The apparently opposing effects of SIRT1 on HIF-1α and HIF--2α could skew cells toward either HIF-1α or HIF-2α transcriptional programs in response to changing metabolic activity in hypoxic tumors. It is possible that other acetylation and deacetylation events regulate HIF activity. For example, in mice, arrest defective 1 (ARD1) was reported to destabilize HIF-1α by acetylating Lys532 [107], an event that is apparently reversed by the recruitment of histone deacetylase 1 (HDAC1) to HIF1α by metastasis-associated protein 1 (MTA1) [108]. Finally, a growing number of reports indicate that HIFα proteins are subject to numerous other post-translational modifications, including sumoylation, S-nitrosylation and neddylation [109,110,111,112,113], although whether any of these modifications differentially regulate HIF-1α and HIF-2α is as yet unknown.

## 6. ROS Generation under Hypoxic Conditions and Its Involvement in HIF-1 Activation

Moderate hypoxic conditions (1–5%) cause a dearth of oxygen, the final electron acceptor of the electron transport system, whereby electrons lose their acceptor and are unable to pass through the cytochrome C-respiratory enzyme chain complex. These electrons then react to produce superoxide (O^2−^) [92,93,114]. Mn-SOD or Cu/Zn SOD in mitochondria convert O^2−^ to hydrogen peroxide, which subsequently leaves the mitochondria to oxidize divalent irons into a trivalent status or to modify the hydroxylase of the HIF-1α subunit, thereby inhibiting the HIF-1α hydroxylation system. Inhibition of the HIF-1α subunit hydroxylation converts it to an activated form. Cells cultured under special conditions produce live ρ0 cells that are deficient in mitochondrial DNA. Intracellular accumulation of HIF-1α protein and HIF-1-dependent gene expression is not observed under experimental conditions even when these ρ0 cells are exposed to 1% hypoxia. Furthermore, when the cell is exposed to a hypoxic environment, ROS generate from the mitochondria. The theory that the true hypoxia sensor is in mitochondria is based on the experimental fact that ROS are produced, and that HIF activation occurs in a ROS-dependent manner [90,91,115,116].

There is also a counterargument to this opposition scheme. The deletion of PHDs causes losses in HIF-1 regulation to become insensitive to oxygen partial pressure control. There has been no counter-example of this phenomenon until now. However, this only confirms that hydroxylation modification is an essential process in HIF-1α regulation by oxygen partial pressure and does not indicate that PHD is only a bona fide oxygen partial pressure sensor. Several reports have indicated that, in ρ0 cells, HIF-1α protein expression is regulated in an oxygen partial pressure-dependent manner [93].

## 7. Response to Intermittent Hypoxia

Hypoxia is not exclusively persistent. Intermittent hypoxia may normally occur in vivo. Persistent hypoxemia, characteristic of congenital heart disease, causes pulmonary arteries to contract and causes pulmonary hypertension. On the other hand, intermittent hypoxia occurring in excess of 100 times per night, such as during sleep apnea, is known to induce an increase in systemic blood pressure. A process that begins with ROS has been proposed as the mechanism underlying the onset of the above condition [117]. Intermittent hypoxia accompanied by recurrent oxygenation is typical of the sleep apnea syndrome. Generated ROS cause an increase in the intracellular calcium ion (Ca^2+^) concentration. The increase in Ca^2+^ activates HIF-1, that induces NADPH oxidase 2 (NOX2) expression. Thus, the current study focused on ROS production. On the other hand, ROS suppress the activation of HIF-2, which induces the expression of Mn-SOD, which has the effect of eliminating ROS. Sustained ROS production suppresses heme oxygenase-2 (HO-2) activity, promotes the production of carbon monoxide and hydrogen sulfide, and stimulates sympathetic nerves in order to facilitate the entry of ROS into the circulatory system [118,119,120,121]. In this manner, the response of the body to continuous hypoxia is different from its response to intermittent hypoxia [122].

## 8. Induction of HIF-1 Activity under Non-Hypoxic Conditions

It is reported that deletion and mutations of tumor suppressor genes, such as p53, PTEN, and VHL, confer HIFs activation even under non-hypoxic conditions [123,124,125,126,127]. Receptor stimulation by growth factors, such as HER2, insulin-like growth factor 1, and insulin, induced HIF-1 activation under non-hypoxic conditions [128,129,130]. Muscarinic receptors and nicotinic receptors are also known [131]. Furthermore, there is a report that prostaglandin E1 and E2, which are inflammatory mediators, also activate HIF-1 in an EP receptor-dependent manner [132,133]. PI3K (phosphoinositide 3-kinase) is activated by a signal from the receptor; a signal is transmitted to mTOR (mammalian target of rapamycin) and S6 kinase via Akt, and translation of the HIF-1α protein from mRNA is enhanced [134,135]. As a result, HIF-1α protein accumulates in the cell and activates HIF-1. In parallel with this pathway, translation enhancement via MAPK (mitogen-activated protein kinase) has been reported. Treatment with cigarette smoke extract and smoking resulted in the expression of HIF-1α protein and promoted the expression of pro-inflammatory factors such as VEGF and HO-1 in established cell lines derived from the alveolar epithelium and in vivo mice. Smoking also promotes the expression of the Rtp801 (REDD1) gene and matrix metalloproteinase (MMP), which are attracting attention as molecules that promote alveolar damage and changes in emphysema [136]. This activation requires the production of ROS. In other words, the generated ROS may lead to the activation of HIF-1 regardless of the oxygen partial pressure of the environment.

**Figure 6:**
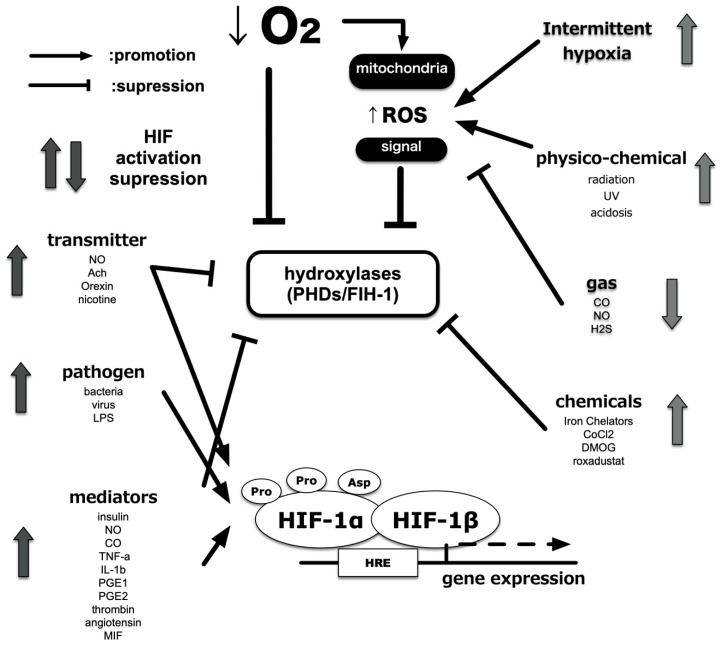
Alternatives to HIF activity regulation. In addition to the partial pressure of O_2_, other factors related to HIF activity have been identified. Various intracellular processes affect HIF activity independently of O_2_ partial pressure and via crosstalk with low O_2_ signals. In addition, certain intracellular signals result in enhanced translation of HIF-1α protein and promote HIF-1 activation.

Cytokines and chemokines mediate inflammation. These regulate phagocyte activity and recruit leukocytes. These also cause fever, which is a typical symptom of local and systemic inflammation. Both proinflammatory cytokines, TNF-α and IL-1β, reportedly activate HIF-1 [137,138,139]. TNF-α and IL-1β activate HIF-1 via multiple pathways, including ROS and nitric oxide (NO) production and phosphoinositide 3-kinase (PI3K) and/or nuclear factor κB (NF-κB) activation. Moreover, mediators of the inflammatory microenvironment, including adenosine and lipopolysaccharide (LPS), also activate HIF-1. HIF-1α protein expression is induced following stimulation of the adenosine receptor and toll-like receptor 4 (TLR-4) in a PI3K-dependent and ROS-dependent manner [140]. Increases in protein translation shift the balance by overwhelming the degradation system, resulting in increased HIF-1α protein. The steady-state of HIF-1α expression is mainly determined by the hydroxylation-mediated degradation system in the proteasome (Figure 6).

NO, regardless of whether exogenously added or endogenously produced, increases HIF-1α protein and causes transactivation of HIF-1 even under normoxic conditions [141,142,143]. NO decreases PHD activity and inhibits HIF-1α ubiquitination, suggesting that hypoxia and NO adopt synergistic intracellular pathways to stabilize and increase HIF-1α [144,145]. Another study using the NO donor, NOC-18, reported that NO increases PI3K activity and HIF-1α protein translation in a mTOR-dependent manner, even under normoxic conditions [146,147]. By contrast, when HIF-1α expression was analyzed under conditions of 1% O_2_, treatment with a NO donor, DETA/NO, suppressed the accumulation of the HIF-1α protein by affecting the mitochondrial electron transfer chain [148,149]. This paradox can be explained by the observation that NO competes with O_2_ for binding to mitochondrial cytochrome oxidase, which consumes most of the O_2_ within the cell [148,149]. Inhibition of PHDs by NADPH oxidase-mediated ROS production is proposed as the underlying regulatory principle. In fact, exogenous H_2_O_2_ also induces the expression of HIF-1α protein and increases HIF-1 activity. It is reported that ROS oxidize Fe (II) at the catalytic site of PHDs, thus blocking such activity [149]. Another possibility is that ROS inhibits PHDs via the oxidation of active site amino acids [148]. These possibilities indicate that an increase in ROS during inflammation may contribute to HIF-1α accumulation and its activation.

### A Close Interaction between NF-κB and HIFs

HIFs play a crucial role in the cellular hypoxic response. However, these are not the only molecules that regulate sensitivity to environmental O_2_. At present, over 20 different transcription factors have been reported as mediating different forms of hypoxic response, either directly or indirectly.

Members of the nuclear factor κB (NF-κB) family of transcription factors regulate inflammation and orchestrate immune response and tissue homeostasis. Members of this family interact with members of the PHD–HIF pathway in a manner that links inflammation to hypoxia [150,151,152]. Studies conducted on mouse inflammatory bowel disease models indicate that PHDs play a regulatory role in the antiapoptotic effect exerted by NF-κB during intestinal inflammation [153]. Hypoxia during intestinal ischemia- reperfusion activates NF-κB in intestinal epithelial cells, which increase TNF-α production but also simultaneously attenuate intestinal epithelial apoptosis. Additional interaction between hypoxia and inflammation involves the IκB kinase complex, a regulatory component of NF-κB, which regulates HIF-1α transcription before and during inflammation. Hypoxia amplifies the NF-κB pathway by increasing the expression and signaling of TLRs, which in turn enhances the production of antimicrobial factors and stimulates phagocytosis, leukocyte recruitment, and adaptive immunity.

NF-κB activity is regulated by inhibitors of NF-κB (IκB) kinases (IKKs), mainly IKKβ, which induce phosphorylation-dependent degradation of IκB inhibitors in response to infectious or inflammatory stimuli. HIF mediates NF-κB activation in neutrophils under anoxic conditions and promotes the expression of NF-κB-regulated cytokines in macrophages stimulated by LPS in a TLR4-dependent manner [138,154]. Interestingly, hypoxia itself can stimulate NF-κB activation by inhibiting prolyl hydroxylases that negatively modulate IKKβ catalytic activity [150].

NF-κB contributes to increased HIF-1α mRNA transcription under hypoxic conditions. Activation of HIF-1α transcription by bacteria or LPS under normoxic as well as hypoxic conditions has recently been reported by a study using mice deficient in IKKβ [152]. Macrophages infected with Gram-positive or Gram-negative bacteria, and mice subjected to hypoxia, exhibited a marked defect in HIF-1α expression, following the deletion of the gene encoding IKKβ [152]. These results confirmed that transcriptional activation of HIF-1α by IKKβ-responsive NF-κB is a crucial precursor to post-transcriptional stabilization and accumulation of the HIF-1α protein.

## 9. Pathophysiological Role of HIFs in Human Diseases

### 9.1. Kidney and Iron Metabolism

Kidneys perform a large amount of glomerular filtration and reabsorb approximately 99% of urine via transporters. This process requires energy and therefore results in ATP consumption. Thus, in order to meet the high demand for energy, kidneys consume a large amount of oxygen [53,155]. In fact, the kidney is in such a low oxygen concentration state that HIF is activated even under normoxic conditions [156,157]. Renal tubule tissues, which extend from the glomerulus to the collective duct in the kidney, undergo resorption and secretion of the glomerular filtrate. The renal tubule is surrounded by capillaries that extend from the glomerulus and reabsorbs most of the water and inorganic salts of the raw urine components discharged into the Bowman’s sac via the renal corpuscles. Even at the highest cortical surface layer, O_2_ partial pressure is between 40 and 60 mmHg but is kept as low as 15 mmHg in the medulla under physiological conditions. Reportedly, a decrease in oxygen partial pressure plays an important role in the progression of acute and chronic renal disorders [156,158]. For example, in contrast agent nephropathy, the hypoxic state that occurs due to an increase in the reabsorption of solute, and a decrease in blood flow in the medulla, plays a critical role in the development of renal failure. An animal experiment indicated that administeration of diuretics to suppress Na^+^ reabsorption during acute renal failure reduced oxygen consumption and increased oxygen partial pressure, thereby alleviating kidney damage [159,160]. Angiotensin II caused a marked decrease in oxygen partial pressure during chronic renal failure, increased oxidative stress, decreased NO, and changed oxygen consumption of mitochondria causing relative hypoxia [161]. Thus, the hypoxic state is involved in the etiology of renal failure [53,155].

EPO, a major target gene of HIFs, is produced in fibroblast cells (renal EPO producing cells) in the renal cortex in a HIF-2-dependent manner [52]. Since HIF-2α is decomposed by hydroxylation in the presence of oxygen molecules, an inhibitor of this hydroxylase domain-containing protein (PHD) has been developed as a new hematopoietic stimulator (ESA). PHD inhibitors may show potential benefits in terms of cost and invasiveness compared to conventional EPO formulations. In fact, the HIF-PH inhibitor, roxadustat, is commercially available in Japan.

When hematopoiesis in the bone marrow increases resulting in an increased demand for iron, the expression of various genes involved in iron utilization and absorption is induced. Because many of these inductions are brought about by HIF-1 and HIF-2, activation of HIFs is thought to accelerate the hematopoietic response via the efficient use of iron [162,163]. Hepcidin is important as an internal defense mechanism that suppresses excessive iron uptake [164,165,166,167]. Hepcidin, produced in hepatocytes, is a small peptide with a molecular weight of 2.8 kDa. It promotes cellular uptake and degradation through phosphorylation of the iron excretion pump ferroportin and suppresses iron absorption, storage, release, and recycling. Hepcidin, which is induced by BMP-SMAD and IL6-STAT3 signaling, causes iron refractory anemia during inflammation. Patients undergoing hemodialysis therapy have high blood hepcidin levels due to various factors such as chronic inflammation and infection, which contributes to the anemia refractory to EPO. On the other hand, hypoxia suppresses hepcidin expression, thereby promoting the uptake and release of iron from reticuloendothelial cells. Although the molecular mechanism underlying this reaction remains unknown, recent studies have shown that HIF directly suppresses hepcidin via bone marrow hematopoiesis by EPO. Furthermore, HIF degrades hemojuvelin, a cofactor of the BMP receptor, via transcription of matriptase-2, and suppresses hepcidin expression. The mechanism of hepcidin suppression by hypoxia at the molecular level as well as the individual level is becoming increasingly clear. Treating anemia via HIF activation is expected to effectively improve anemia associated with chronic inflammation, which is a condition often seen in hemodialysis patients [168,169,170].

### 9.2. Cancer Progression

In solid cancerous tumor tissue, cancer cells grow at a very high rate. In contrast, the rate of angiogenesis inside the tumor is slow, the tumor vessels are extremely fragile and meandering, and frequent occlusion and blood regurgitation occur [171,172]. These result in hypoxic regions where tumor tissue is not supplied with sufficient oxygen, which is known to activate HIFs. In addition, VHL deletion in clear cell carcinoma of the kidney and many other HIF may be activated independently of hypoxia, such as activation of the phosphoinositide 3-kinase (PI3K)/Akt pathway [173]. There is a positive correlation between HIFs expression and poor prognosis.

HIFs contribute to the adaptation to hypoxia in metabolic reprogramming and angiogenesis in cancer cells as well as normal cells, and in particular, cancer cells generate ATP by glycolysis even under normal oxygen conditions [173]. Many studies have shown that HIF-1 is deeply involved in this phenomenon (Warburg effect) [174,175,176]. Furthermore, cancer tissues do not have sufficient blood flow. Thus cancer cells are also undernutrition. Not only do cancer cells adapt to their hypoxia and hyponutrition environment through HIFs, but they also change the cells that make up cancer tissues [177,178]. In recent years, the relationship between cancer metastasis and HIFs has been elucidated [179]. Metastasis consists of many steps, and epithelial–mesenchymal transition (EMT) is a particularly essential phenomenon [180,181]. HIFs induces the expression of the transcription factor TWIST, which regulates EMT, and induces nuclear translocation of the EMT regulator SNAIL. In addition, HIFs cooperates with transforming growth factor (TGF)-β to produce Sma and Mad Related Activates the Family (SMAD) pathway and causes EMT, including a decrease in E-cadherin. In addition, in breast cancer, HIF-1-induced angiopoietin-related protein 4 (ANGPTL4) inhibits adhesion between vascular endothelial cells and promotes cancer extravasation. In addition, HIF-1 produces lysyl oxidase (LOX) and LOX-like proteins from cancer cells. It has also been reported that the outer matrix is remodeled to promote cancer cell invasion [182,183].

### 9.3. Lactate and Hypoxia Response

High serum lactate (> 2 mmol/L) constitutes a diagnostic criterion for septic shock in the “International consensus definition of sepsis and septic shock in third edition (Sepsis-3)”. The established consensus is that lactic acidosis indicates a negative mismatch between the demand for oxygen and its supply in the peripheral tissues. However, this consensus has now been revised. A classification of hyperlactatemia was proposed: With tissue oxygenation deficiency and without tissue oxygenation deficiency as Type A and Type B, respectively [184].

Propofol is widely used as intravenous anesthesia. Propofol infusion syndrome (PRIS) is a manifestation of propofol toxicity [185,186,187]. Propofol infusion syndrome is usually detected in those who have been administered high doses of propofol for an extended time. When propofol reaches toxic levels, it uncouples oxidative phosphorylation in the mitochondrial electron transport chain, causing severe acidosis [186]. Propofol also inhibits carnitine palmitoyltransferase, which is involved in fatty acid metabolism. Fatty acid accumulation can lead to arrhythmia and poor energy availability [26]. An imbalance in demand for energy and its supply may lead to organ dysfunction. The authors used an extracellular flux analyzer that measures the oxygen consumption rate (OCR) of cells and extracellular pH change (ECAR), which reflects lactic acid production [27,188]. Exposure to hypoxia for approximately 2 h may lead to metabolic reprogramming, resulting in switching from oxidative phosphorylation to glycolysis in a HIF-1 dependent manner [27,188]. OCR is suppressed and ECAR is enhanced. On the other hand, propofol, which is used for anesthesia and sedation, also suppresses oxygen consumption and stimulates lactic acid production in approximately 4 h when cells are exposed to a concentration of approximately 25 μM. Drug-induced mitochondrial disorder is an example of promoting lactic acid production. In addition, clinical concentrations of propofol facilitate the conversion of cellular metabolic mode easily when mitochondrial function is suppressed for unknown reasons.

### 9.4. Cardiac Hypertrophy and Heart Failure

Takeda and a colleague established a mouse cardiac fibrosis and remodeling model induced by narrowing the transverse aorta. They found that myocardial tissue was hypoxic and that there was an accumulation of macrophages in the hypoxic regions of the heart, by examination via a phosphorescent probe as well as an analysis based on flow cytometry [189,190]. Furthermore, by analyzing mice with suppressed HIF-1α signaling of macrophages, they found that HIF-1α signaling induced macrophage accumulation in the heart [191]. As fibrosis of mice hearts increased with suppressed HIF-1α signaling, it was believed that macrophage accumulation in the heart suppressed fibrosis [190,192]. Next, they investigated the mechanism by which macrophage accumulation in the heart inhibited fibrosis, and reported that macrophage associated HIF-1α signaling produced oncostatin M, a cytokine, which inhibited the activation of cardiac fibroblasts by suppressing TGF-β/Smad signaling, thereby suppressing the fibrosis of myocardial tissue. This revealed that macrophage accumulation in the heart suppresses excessive fibrosis of the heart by producing oncostatin M [190]. Heart fibrosis prevention effect of oncostatin M may indicate a new therapeutic target for heart fibrosis and heart failure. The central fructose-metabolizing enzyme is ketohexokinase (KHK), which exists in two isoforms: KHK-A and KHK-C, generated through mutually exclusive alternative splicing of KHK pre-mRNAs. It was demonstrated that splice factors as HIF-1α targets, which prompted the analysis of their RNA targets, unveiling mechanistic and functional linkages between HIF-1α, splice factor 3b subunit 1 (SF3B1), KHK-C splice isoform production and fructose metabolism in cardiac hypertrophy. Myocardial hypoxia enhances fructose metabolism in human and mouse models of pathological cardiac hypertrophy through HIF-1α activation of SF3B and SF3B1-mediated splice switching of KHK-A to KHK-C [193]. Thus, HIF-1 has been shown to have important roles under various pathways for hypertrophy and heart failure.

### 9.5. Placenta Formation and Oxygen Tension-Pregnancy Induced Hypertension and Intrauterine Growth Retardation

The placenta is a target for the elucidation of the pathogenesis of pregnancy-induced hypertension (PIH), intrauterine growth retardation (IUGR) and therapeutic intervention [194,195]. The etiology of PIH was presumed to be “toxins” produced in the placenta and other organs of pregnant women, which caused hypertension and proteinuria, leading to the onset of PIH [196]. Lately, impairment of placental circulation has come to be considered as the new etiology. Histological findings closely associated with PIH have indicated its causes as the shallowness of infiltration of the trophoblast into the muscle layer and the smallness of the vasculature in the placenta of PIH cases. In this context, the association between hypoxia and trophoblast proliferation/infiltration was stressed. Reportedly, infiltration of the trophoblast and its proliferation are enhanced under hypoxic conditions. This suggests that oxygen metabolism may be involved in the etiology of PIH, IUGR, or eclampsia, and therefore considered a target in the treatment of these conditions [197].

Implantation disorders are a major issue in reproductive medicine, but effective diagnosis and treatment have not yet been established. Implantation is achieved by embryos entering the uterus and adhering to the endometrium (embryo adhesion) and the process of embryos entering the endometrium (embryo infiltration) [198,199]. Although precise interaction between the uterus and the embryo is supposedly essential for the establishment of implantation, details of the underlying mechanism remain unknown. It is demonstrated by using reports that it is HIF-2, and not HIF-1, that exclusively acts in the endometrium to regulate the process of embryonic invasion [199,200]. Due to the role played by endometrial stromal HIFs, the endometrial luminal epithelium is peeled off to expose the endometrial stroma, enabling easier entry of the embryo into the endometrial stroma. Simultaneously, they also clarified that the endometrial stroma was in close contact with the embryo and that the embryo was laboring to survive. As a result, the mechanism of implantation occurring in the uterus has been elucidated, and the cause of infertility due to implantation failure may be clarified in the near future. It is hoped that future examinations of the effect of HIFs on human endometrium may lead to the development of new diagnostic and therapeutic methods for implantation disorders.

### 9.6. Immunity

It has been reported that stimulation of the T-cell receptor/CD3 antigen complex may also activate HIF-1 in T-cell lymphocytes [201,202]. This suggests that not only innate immunity but also acquired immunity elicited by antigens are affected by oxygen partial pressure and HIF-1 [139,203]. Cellular glucose metabolism and fat metabolism play a major role in the process of naive T-cell differentiation into helper T cells17/regulatory T Treg cells, which are vital for regulating the immune system. Cells producing IFN-γ, which modulates processes associated with cellular immunity, such as viral exclusion, are produced by different cytokines. Th2 cells produce IL-4, which regulates processes related to humoral immunity, such as the elimination of parasites, and Th17 cells produce IL-17 and Treg cells that suppress the immune system. Of these, it has been suggested that the RAR-related orphan receptor (ROR) γt, the main regulator of Th17 cell differentiation, produces IL-17 with the cooperation of HIF-1α. On the other hand, under Th17 cell-inducing conditions, ubiquitinated HIF-1α binds to Foxp3, a major regulator of Treg cell differentiation, and is degraded along with Foxp3 in the proteasome. These results suggest that HIF-1 regulates the balance between Th17 and Treg cells. These findings indicate the importance of oxygen metabolism to many processes, including the maintenance of antibody quality and class switching, which even applies to processes such as B-cell activation.

### 9.7. Role of Hypoxia on Autophagy and Cellular Damage

Autophagy is a system in which lysosomes degrade proteins and organelles in the cytoplasm. The ubiquitin–proteasome system is also used to degrade intracellular components, but the ubiquitin–proteasome system is used to degrade relatively short-lived proteins [204,205,206]. Intracellular proteins and organelles are degraded by autophagy when they are no longer needed or as needed, and their components are reused in the cells. Autophagy activity is low in normal cells but becomes upregulated and activated when cells are exposed to an environment such as starvation or hypoxia [207]. Autophagy is a tightly controlled pathway, an important and clever system built into the cell to sustain its life under stress.

HIF-1-induced B-cell lymphoma 2 (BCL2)/adenovirus E1B19kDa protein-interacting protein 3 (BNIP3) competes with BCL2 to release Beclin1 involved in autophagosome formation. BNIP3 promotes autophagy by binding to and inhibiting Ras homolog enriched in brain (Rheb), which is important for activation of the mTOR pathway [208,209,210]. As a result, mitophagy (mitochondrial autophagy) is triggered, and dysfunctional mitochondria are removed. A mechanism for maintaining ATP production and a mechanism for adapting to a hypoxic environment by suppressing ATP consumption have been reported. Regulated in development and DNA damage response 1 (REDD1) induced by HIF-1 inhibits the binding of tuberous sclerosis protein 2 (TSC2), a suppressor of mTOR, to 14–3-3 protein [211]. The released TSC2 suppresses ATP consumption by suppressing the mTOR pathway and reducing gene translation efficiency [209,210].

## 10. Conclusions

In this review, I described the current state of researches on hypoxia response and oxygen metabolism in a living body and explained the relationship between various biological phenomena and the HIF system.

## Figures and Tables

**Table 1:** Enzymatic properties of HIF-α hydroxylases and type I collagen hydroxylase.

		Substrate		Km (µM)			
Gene	Protein	Pro-402	Pro-564	O_2_	α-ketoglutarate	Ascorbate	Fe(II)
*EGLN1*	PHD2/HPH-2	+	+	230	60	170	0.03
*EGLN2*	PHD-1/HPH-3	+	+	250	60	180	0.1
*EGLN3*	PHD3/HPH-1	−	+	230	55	140	0.03
*HIF1AN*	FIH-1	Asp-803		90	25	260	0.5
*P4HA1*	C-P4H-I	collagen		40	20	300	
